# Supplementary material for: Dynamical modulation of hippocampal replay through firing rate adaptation
Source: Nat Commun. 2026 Jan 20;17:1282. doi: 10.1038/s41467-025-68042-3 (PMC12868804; doi:10.1038/s41467-025-68042-3)
Supplement: Supplementary file 1 — Supplementary Information [file 41467_2025_68042_MOESM1_ESM.pdf]

Dynamical modulation of hippocampal replay through firing rate adaptation Supplementary Information

Zilong Ji[1, 2, 5, †], Tianhao Chu[1,2,†], Xingsi Dong[2, †], Changmin Yu[6],

Daniel Bush[7], Neil Burgess[5,8,*], Si Wu[1,2,3,4, *].

[1]School of Psychological and Cognitive Sciences, Peking University, China.

[2] Peking-Tsinghua Center for Life Sciences, Academy for Advanced Interdisciplinary Studies, Peking University, China.

[3]PKU-IDG/McGovern Institute for Brain Research, Peking University, China.

[4]Center of Quantitative Biology, Peking University, China.

[5]Institute of Cognitive Neuroscience, University College London, UK

[6]Computational and Biological Learning Lab, Department of Engineering, University of Cambridge, UK

[7]Department of Neuroscience, Physiology and Pharmacology, University of College London, UK.

[8]UCL Queen Square Institute of Neurology, University College London, UK

[†]: These authors contributed equally to this work.

[*]: Correspondence: n.burgess@ucl.ac.uk; siwu@pku.edu.cn

1 Stability analysis of the bump activity

We are now considering a more complex case by including the dynamics of firing rate adaption. Thus, the stability analysis given in Methods 4.4 is a special case when $m_\beta = 0$. Since the external sensory-motor dependent input is not considered in the analysis here, we simply set $m_\beta = m$ below. Specifically, the network dynamics is:

$$\tau_u \frac{dU(x,t)}{dt} = -U(x,t) + \rho \int_{-\infty}^{\infty} J(x,x') r(x',t)\, dx' - V(x,t) + I^{ext}(x,t), \quad (1)$$

$$\tau_v \frac{dV(x,t)}{dt} = -V(x,t) + mU(x,t), \quad (2)$$

$$r(x,t) = \frac{U(x,t)^2}{1 + k\rho \int_{-\infty}^{\infty} U(x',t)\, dx'}, \quad (3)$$

and the assumed network state is written as:

$$\overline{U}(x,t) = A_u \exp\left\{-\frac{[x-z(t)]^2}{4a^2}\right\}, \quad (4)$$

$$\overline{V}(x,t) = A_v \exp\left\{-\frac{[x-z(t)+s(t)]^2}{4a^2}\right\}, \quad (5)$$

$$\overline{r}(x,t) = A_r \exp\left\{-\frac{[x-z(t)]^2}{2a^2}\right\}. \quad (6)$$

Substituting Eqs.(4,5,6) into the network dynamics Eqs.(1,2,3), we get

$$A_r = \frac{A_u^2}{1 + k\rho\sqrt{2\pi} a A_u^2}, \quad (7)$$

$$\tau \left[A_u \frac{x-z}{2a^2}\frac{dz}{dt} + \frac{dA_u}{dt}\right] \mathcal{N}(z,2a) = (-A_u + \frac{\rho J_0}{\sqrt{2}} A_r)\mathcal{N}(z,2a) - A_v \mathcal{N}(z-s,2a) + I^{ext}(x,t), \quad (8)$$

$$\tau_v \left[A_v \frac{x-z+d}{2a^2}\frac{d(z-s)}{dt} + \frac{dA_v}{dt}\right] \mathcal{N}(z-s,2a) = -A_v \mathcal{N}(z-s,2a) + mA_u \mathcal{N}(z,2a), \quad (9)$$

where $\mathcal{N}(z, 2a) = exp\left\{-[x-z]^2/4a^2\right\}$.

Since the dynamics of a CANN is dominated by a few motion modes, we therefore simplify the network dynamics by projecting the network dynamics on its dominating motion modes (by projecting a function $f(x)$ onto a mode $u_n(x)$, it means to compute $\int_x f(x)u_n(x)dx$. For the bump $U(x,t)$, the first two motion modes are,

$$u_0(x,t) = \exp\left\{-\frac{[x-z(t)]^2}{4a^2}\right\}, \tag{10}$$

$$u_1(x,t) = [x-z(t)]\exp\left\{-\frac{[x-z(t)]^2}{4a^2}\right\}, \tag{11}$$

and for the bump $V(x,t)$, the first two motion modes are,

$$v_0(x,t) = \exp\left\{-\frac{[x-z(t)+s(t)]^2}{4a^2}\right\}, \tag{12}$$

$$v_1(x,t) = [x-z(t)+s(t)]\exp\left\{-\frac{[x-z(t)+s(t)]^2}{4a^2}\right\}. \tag{13}$$

To do the stability analysis, we simply set $I^{ext}(x,t) = 0$ where the CANN is not driven by sensory-motor dependent location input. Assume that the activity bump is stable, then $dz/dt = 0$, and the discrepancy between them is zero, i.e., $s = 0$. Thus, Eqs. (8)-(9) are simplified as:

$$\tau\frac{dA_u}{dt} = -A_u + \frac{\rho J_0}{\sqrt{2}}A_r - A_v, \tag{14}$$

$$\tau_v\frac{dA_v}{dt} = -A_v + mA_u. \tag{15}$$

Combining with Eq. (7), we get the height value of the activity bumps in the adaptive CANN, which are:

$$A_v = mA_u, \tag{16}$$

$$A_r = \frac{\sqrt{2}(1+m)}{\rho J_0}A_u, \tag{17}$$

$$A_u = \frac{\rho J_0 + \sqrt{\rho^2 J_0^2 - 8\sqrt{2\pi}(1+m)^2 k\rho a}}{4\sqrt{\pi}(1+m)k\rho a}. \tag{18}$$

To analyze the stability of this solution, we calculate the Jacobian matrix at this point, which is:

$$\mathbf{M} = \begin{pmatrix} \frac{1}{\tau}\left(-1+\frac{\sqrt{2}\rho J_0 A_u}{(1+\sqrt{2\pi}k\rho a A_u^2)^2}\right) & -\frac{1}{\tau} \\ \frac{m}{\tau_v} & -\frac{1}{\tau_v} \end{pmatrix} \tag{19}$$

Denote the eigenvalues of the Jacobian matrix $\mathbf{M}$ as λ_1 and λ_2, then the condition for the solution to be stable is that both eigenvalues are negative, which means,

$$\lambda_1+\lambda_2 = \frac{1}{2}\left[-1+\frac{\sqrt{2}A_u J_0\rho}{(1+\sqrt{2\pi}k\rho a A_u^2)^2} - \frac{\tau}{\tau_v}\right] < 0, \tag{20}$$

$$\lambda_1\lambda_2 = \frac{\tau}{\tau_v}\left(m+1-\frac{\sqrt{2}A_u J_0\rho}{(1+\sqrt{2\pi}k\rho a A_u^2)^2}\right) > 0. \tag{21}$$

The above inequalities can be satisfied when

$$0 < k < k_{c_1} \equiv \frac{\rho J_0^2(1+\frac{\tau}{\tau_v})(1+2m-\frac{\tau}{\tau_v})}{8\sqrt{2\pi}a(1+m)^4}, \tag{22}$$

$$0 < k < k_{c_2} \equiv \frac{\rho J_0^2}{8\sqrt{2\pi}a(1+m)^2}. \tag{23}$$

It is easy to check that $k_{c_2} < k_{c_1}$, so the condition for the network to hold activity bump as its stable state is $0 < k < k_{c_2}$. When the firing rate adaptation does not exist in the CANN, i.e., $m = 0$, then the condition of holding activity bump as the stable state for the CANN is $0 < k < k_{c_2} \equiv \rho J_0^2/8\sqrt{2\pi}a$, which is the case we showed in Methods 4.4.

2 Getting the height dynamics of A_u and A_v using the projection method

Here we show how to use the projection method to get the expression of bump height and bump position, respectively. First, we focus on the dynamics of bump height A_u and A_v. We first substitute the assumed bump expressions Eqs. (4)-(6) into the CANN dynamics Eq. (1), which gives the expression of A_u (to prevent the wrapping of expressions, we show the results of left side and right side respectively):

$$
\begin{aligned}
l-side &= \tau \exp\left[-\frac{(\mathbf{x}-\mathbf{z})^2}{4a^2}\right]\frac{\mathrm{d}A_u}{\mathrm{d}t} + \tau_u \frac{A_u(\mathbf{x}-\mathbf{z})}{2a^2}\exp\left[-\frac{(\mathbf{x}-\mathbf{z})^2}{4a^2}\right]\frac{\mathrm{d}\mathbf{z}}{\mathrm{d}t}, \quad (24)\\
r-side &= \left(-A_u + \frac{\rho J_0 A_r}{2}\right)\exp\left[-\frac{(\mathbf{x}-\mathbf{z})^2}{4a^2}\right] - A_v \exp\left[-\frac{(\mathbf{x}-\mathbf{z}+\mathbf{s})^2}{4a^2}\right] + \\
&\quad \sigma_U \xi_U(\mathbf{x}, t). \quad (25)
\end{aligned}
$$

As mentioned in the main text, projecting a function $f(\mathbf{x})$ onto a mode $u(\mathbf{x})$ equals to computing $\int_{\mathbf{x}} f(\mathbf{x})u(\mathbf{x})d\mathbf{x}/\int_{\mathbf{x}} u^2(\mathbf{x})d\mathbf{x}$. Therefore, by projecting both sides onto the motion mode $u_0(\mathbf{x}|\mathbf{z})$, we obtain:

$$
\begin{aligned}
l-side &= \tau_u 2\pi a^2 \frac{\mathrm{d}A_u}{\mathrm{d}t} + \frac{\tau_u A_u}{2a^2}\frac{\mathrm{d}\mathbf{z}}{\mathrm{d}t}\int_{-\infty}^{\infty}\mathrm{d}\mathbf{x}(\mathbf{x}-\mathbf{z})\exp\left[-\frac{(\mathbf{x}-\mathbf{z})^2}{2a^2}\right], \quad (26)\\
r-side &= \left(-A_u + \frac{\rho J_0}{2}A_r\right)2\pi a^2 - A_v \exp\left[-\frac{\mathbf{s}^2}{8a^2}\right]2\pi a^2 + \sqrt{2\pi}a\sigma_U\xi_{U,0}. \quad (27)
\end{aligned}
$$

Note that the part inside the integral in Eq. (26) is an odd function which integrates to zero. Intuitively, this odd function can be regard as the variation of the bump position along the dimension of the bump height, which are orthogonal to each other. In other words, this term has no effect on the dynamics of A_u. Thus, by equating both sides, we obtain,

$$
\tau_u \frac{\mathrm{d}A_u}{\mathrm{d}t} = \left(-A_u + \frac{\rho J_0}{2}A_r\right) - A_v \exp\left[-\frac{\mathbf{s}^2}{8a^2}\right] + \frac{1}{\sqrt{2\pi}a}\sigma_U\xi_{U,0}, \quad (28)
$$

where $\xi_{U,0}$ is obtained by projecting the noise term ξ_U onto the motion mode $u_0(\mathbf{x}|\mathbf{z})$. More concretely, the newly defined noise term is given by,

$$
\xi_{U,0}(t) = \int_{\mathbf{x}} \xi_U(\mathbf{x}, t)u_0(\mathbf{x}|\mathbf{z})\mathrm{d}\mathbf{x}. \quad (29)
$$

It is straightforward to check that

$$
\begin{aligned}
\langle \xi_{U,0}(t)\rangle &= \int_{\mathbf{x}} \langle \xi_U(\mathbf{x}, t)\rangle u_0(\mathbf{x}|\mathbf{z})\mathrm{d}\mathbf{x} = 0, \quad (30)\\
\langle \xi_{U,0}(t)\xi_{U,0}(t')\rangle &= \int_{\mathbf{x}}\mathrm{d}\mathbf{x}\int_{\mathbf{x}'}\mathrm{d}\mathbf{x}'\langle \xi_U(\mathbf{x}, t)\xi_U(\mathbf{x}', t')\rangle u_0(\mathbf{x}|\mathbf{z})u_0(\mathbf{x}|\mathbf{z})\\
&= \delta(t-t'), \quad (31)
\end{aligned}
$$

with δ is a Dirac delta function. Eqs. (30&31) imply that the newly defined noise term $\xi_{U,0}$ is still a Gaussian white noise with zero mean and unit variance, as stated in the main text.

Furthermore, if there is no noise in the network, the separation $\mathbf{s}(t)$ between $U(x, t)$ and $V(x, t)$ has a stationary solution, which is expressed as $\mathbf{s}^2 = a^2(1-\sqrt{m/(\tau/\tau_v)})$. This implies that, as long as the adaptation strength is set around the travelling wave boundary τ/τ_v, $\mathbf{s}^2 \ll a^2$ always holds, which means that $\mathbf{s}^2/a^2 \approx 0$. Therefore, Eq. (28) can be further simplified as,

$$
\tau_u \frac{\mathrm{d}A_u}{\mathrm{d}t} = -A_u - A_v + \frac{J_0 \rho A_r}{2} + \frac{\sigma_U}{a\sqrt{2\pi}}\xi_{U,0}(t), \quad (32)
$$

which corresponds to Eq. (28) in Methods.

To obtain the dynamics of A_v, we substitute the presumed network state (Eq. (4-6)) into the adaptation dynamics (Eq. (2)), which gives,

$$l-side = \tau_v \exp\left[\frac{(\mathbf{x}-\mathbf{z}+\mathbf{s})^2}{4a^2}\right]\frac{\mathrm{d}A_v}{\mathrm{d}t} + \tau_v \frac{A_v(\mathbf{z}-\mathbf{x}-\mathbf{s})}{2a^2}\exp\left[\frac{(\mathbf{x}-\mathbf{z}+\mathbf{s})^2}{4a^2}\right]\frac{\mathrm{d}\mathbf{z}-\mathrm{d}\mathbf{s}}{\mathrm{d}t}, \tag{33}$$

$$r-side = -\tau_v A_v \exp\left[\frac{(\mathbf{x}-\mathbf{z}+\mathbf{s})^2}{4a^2}\right] + [m+\sigma_m \xi_m(\mathbf{x},t)]\, A_u \exp\left[\frac{(\mathbf{x}-\mathbf{z})^2}{4a^2}\right]. \tag{34}$$

Similarly, we project both sides onto the motion mode $u_0(\mathbf{x}|\mathbf{z})$, and obtain the dynamics of A_v,

$$\tau_v \frac{\mathrm{d}A_v}{\mathrm{d}t} = -A_v + mA_u + \frac{\sigma_m A_u}{2a\sqrt{\pi}}\xi_{m,0}(t), \tag{35}$$

which corresponds to Eq. (29) in Methods. Here, $\xi_{m,0}$ is also a Gaussian white noise with zero mean and unit variance.

3 Getting the position dynamics of $\mathbf{z}(t)$ and $\mathbf{s}(t)$ using the projection method

We only present the derivation of the dynamics of $\mathbf{z}(t)$, as the case for $\mathbf{s}(t)$ is similar. To do this, we project both sides the network dynamics Eq. (24)-(25) onto the motion mode $u_1(\mathbf{x}|\mathbf{z})$, we obtain,

$$l-side = \tau\pi a^2 A_u \frac{\mathrm{d}\mathbf{z}}{\mathrm{d}t} + \tau\frac{\mathrm{d}A_u}{\mathrm{d}t}\int_{-\infty}^{\infty}\mathrm{d}\mathbf{x}(\mathbf{x}-\mathbf{z})\exp\left[-\frac{(\mathbf{x}-\mathbf{z})^2}{2a^2}\right], \tag{36}$$

$$r-side = \pi a^2 A_v \mathbf{s}\exp\left[-\frac{\mathbf{s}^2}{8a^2}\right] + a^2\sqrt{2\pi}\sigma_U \xi_{U,1}(t). \tag{37}$$

Note that the part inside the integral in Eq. (36) is also an odd function which integrates to zero. Thus, by equating both sides and utilizing the fact that $\mathbf{s}^2/a^2 \approx 0$, we obtain,

$$\tau_u \frac{\mathrm{d}\mathbf{z}}{\mathrm{d}t} = \frac{A_v}{A_u}\mathbf{s} + \sqrt{\frac{2}{\pi}}\frac{\sigma_U}{A_u}\xi_{U,1}(t), \tag{38}$$

which corresponds to Eq. (30) in Methods. Here, $\xi_{U,1}$ is obtained by projecting the noise term ξ_U to the motion mode $u_1(\mathbf{x}|\mathbf{z})$, i.e.,

$$\xi_{U,1}(t) = \int_{\mathbf{x}} \xi_U(\mathbf{x},t)\, u_1(\mathbf{x}|\mathbf{z})\mathrm{d}\mathbf{x}. \tag{39}$$

It is straightforward to check that

$$\langle \xi_{U,1}(t)\rangle = \int_{\mathbf{x}} \langle \xi_U(\mathbf{x},t)\rangle\, u_0(\mathbf{x}|\mathbf{z})\mathrm{d}\mathbf{x} = 0, \tag{40}$$

$$\begin{aligned}\langle \xi_{U,1}(t)\xi_{U,1}(t')\rangle &= \int_{\mathbf{x}}\mathrm{d}\mathbf{x}\int_{\mathbf{x}'}\mathrm{d}\mathbf{x}'\langle \xi_U(\mathbf{x},t)\xi_U(\mathbf{x}',t')\rangle\, u_1(\mathbf{x}|\mathbf{z})\,u_1(\mathbf{x}|\mathbf{z}) \\ &= \delta(t-t').\end{aligned} \tag{41}$$

This indicates that the newly defined noise term $\xi_{U,1}$ is still a Gaussian white noise with zero mean and unit variance, as stated in the main text.

Similarly, we can obtain the dynamics of $\mathbf{s}(t)$ by projecting Eq.(33-34) onto $u_1(\mathbf{x}|\mathbf{z})$, which gives,

$$\tau_v \frac{\mathrm{d}\mathbf{s}}{\mathrm{d}t} = \left(\frac{\tau_v A_v}{\tau_u A_u} - \frac{mA_u}{A_v} - \frac{\sigma_m A_u \xi_{v,0}}{2A_v\sqrt{\pi}a}\right)\mathbf{s} + \frac{\tau_v\sigma_U}{\tau_u A_u}\sqrt{\frac{2}{\pi}}\xi_{U,1} - \frac{\sigma_m A_u}{A_v}\sqrt{\frac{1}{2\pi}}\xi_{v,1}. \tag{42}$$

This corresponds to Eq. (31) in Methods. Here, $\xi_{m,1}$ is also a Gaussian white noise with zero mean and unit variance.

4 Getting the probability distribution of $\mathbf{s}(t)$

In this section, we present the detailed derivation of the stationary distribution of $\mathbf{s}(t)$. The dynamics of $\mathbf{s}(t)$ (see Eq. (35) in Methods) is as follows,

$$\tau_v \frac{\mathrm{d}s_i}{\mathrm{d}t} = -(\mu + \gamma\xi_v)s_i + \sigma_s\xi_{s_i}, \quad i = \{1,2\}. \tag{43}$$

Here, $\mu = 1 - m\tau_v/\tau$ is the normalized distance to the travelling wave boundary, and $\sigma_m/\left(2\sqrt{\pi}am\right)\xi_{v,0}(t)$ is the noise-to-strength ratio, as defined in the main text. The two variables s_1 and s_2 are the components of $\mathbf{s}$ in the two-dimensional space, respectively. Without loss of generality, we only study one component, and for clearance, we denote $s_i(t)$ and $\xi_{s_i}(t)$ as $s(t)$ and $\xi_s(t)$, respectively.

When the noise-to-strength ratio $\gamma = 0$, the dynamics of $\mathbf{s}(t)$ degenerates to the Ornstein–Uhlenbeck (OU) process, and the stationary distribution of s can be solved as,

$$p^{st}(s) = \sqrt{\frac{\mu}{\pi\sigma_s^2}} exp\left[-\frac{\mu s^2}{\sigma_s^2}\right]. \tag{44}$$

When $\gamma > 0$, the drift term in the dynamics of $\mathbf{s}(t)$ is affected by the multiplicative noise ξ_v. Utilizing Itô calculus, we can rewrite Eq.(43) as a first order difference equation,

$$\begin{aligned} s(t+\mathrm{d}t) &= s(t) + \int_t^{t+\mathrm{d}t}\left(-\frac{\mu s(t')}{\tau_v} + \frac{\gamma s(t')}{\sqrt{\tau_v}}\xi_v(t') + \frac{\sigma_s}{\sqrt{\tau_v}}\xi_s(t')\right)\mathrm{d}t', & (45)\\ &= s(t) - \frac{\mu s(t)}{\tau_v}\mathrm{d}t + \frac{1}{\sqrt{\tau_v}}\left(-s(t)\gamma\mathrm{d}t\overline{\xi_v} + \sigma_s\mathrm{d}t\overline{\xi_s}\right), & (46)\end{aligned}$$

where $\mathrm{d}t\overline{\mu}$ and $\mathrm{d}t\overline{\xi_s}$ are the Ito prescriptions in the limit of $\mathrm{d}t \to 0$.

To derive the Fokker-Planck equation, we adopt a smooth trial function $R(s)$ proposed by Rivers (1), and compute its average value at time t, which is expressed as,

$$\langle\langle R(t)\rangle\rangle = \int R(s)p(s,t)\mathrm{d}s, \tag{47}$$

where $p(s,t)$ is the distribution of $s(t)$ at time t. Consider the evolution of the average value of $R(t)$ in a short interval dt at time t, which is given by,

$$\langle\langle R(t+\mathrm{d}t)\rangle\rangle = \left\langle \int R\left(s - \frac{\mu s}{\tau_v}\mathrm{d}t + \frac{1}{\sqrt{\tau_v}}\left(-s\gamma\mathrm{d}t\overline{\xi_m} + \sigma_s\mathrm{d}t\overline{\xi_s}\right)\right)p(s,t)\mathrm{d}s\right\rangle. \tag{48}$$

With the first-order Taylor-series expansion of the trial function $R(\cdot)$ at s, the right side of Eq.(48) is expressed as,

$$\begin{aligned}&\left\langle \int ds\; p(s,t)R\left(s - \frac{\mu s}{\tau_v}\mathrm{d}t + \frac{1}{\sqrt{\tau_v}}(-s\gamma\mathrm{d}t\overline{\xi_m} + \sigma_s\mathrm{d}t\overline{\xi_s})\right)\right\rangle = \\ &\left\langle \int \mathrm{d}s\; p(s,t)\left[R(s) + \mathrm{d}tR'(s)\left(-\frac{\mu}{\tau_v}s\right) + \mathrm{d}tR''(s)\left(\frac{\sigma_s^2+\gamma^2 s^2}{2\tau_v}\right)\right]\right\rangle. \qquad (49)\end{aligned}$$

Note that the left side of Eq.(48) corresponds to the partial derivative of $p(s,t)$ with respect to t, and the right side of Eq.(49) corresponds to the partial derivative of $p(s,t)$ with respect to s. Thus, we can achieve the following Fokker-Planck expression of the distribution of $s(t)$,

$$\frac{\partial p(s,t)}{\partial t} = -\frac{\partial}{\partial s}\left(-\frac{\mu}{\tau_v}sp(s,t)\right) + \frac{\partial^2}{\partial s^2}\left(\frac{\sigma_s^2+\gamma^2 s^2}{2\tau_v}p(s,t)\right). \tag{50}$$

It describes the time evolution of the distribution of $s(t)$. The stationary distribution of $p^{st}(s)$ is achieved when

$$-\frac{\mu}{\tau_v}sp^{st}(s) = \frac{\mathrm{d}}{\mathrm{d}s}\left(\frac{\sigma_s^2+\gamma^2 s^2}{2\tau_v}p^{st}(s)\right). \tag{51}$$

Thus, we obtain the stationary solution $p^{st}(s)$ as,

$$p^{st}(s) = c_0 \left(\sigma_s^2 + \gamma^2 s^2\right)^{-(1+\mu/\gamma^2)}, \tag{52}$$

where c_0 is a normalization constant. This corresponds to Eq. (36) in Methods.

5 Getting the probability distribution of $\|\Delta\mathbf{z}\|$

According to the general Fokker-Planck expression of the distribution of $s(t)$ (Eq. (50)), we have,

$$\langle s(t+\delta t)\rangle = \langle s(t)\rangle \exp\left[-\frac{\mu\delta t}{\tau_v}\right], \tag{53}$$

$$\langle s(t+\delta t)s(t)\rangle - \langle s(t+\delta t)\rangle\langle s(t)\rangle = \left(\langle s^2(t)\rangle - \langle s(t)\rangle^2\right)\exp\left[-\frac{\mu\delta t}{\tau_v}\right]. \tag{54}$$

The exponential terms on the right side in the above equations indicate that $s(t)$ relaxes exponentially fast to the stationary distribution Eq. (52) on the time scale of $\delta t \gg \tau_v$. Lubashevsky et al. (2) demonstrated that the displacement of $z(t)$ in δt (denote as Δz hereafter) is dominated by the maximal value of s in this time interval (denote as s_{max} hereafter), that is, $\Delta z(\tau_v) \sim s_{max}\tau_v$. Due to the temporal independence of $s_{max}(t)$ when $\delta t \gg \tau_v$, the value of Δz can be acquired by sampling from the stationary distribution of $s_{max}(t)$, i.e. the stationary distribution of $s(t)$. Thus, substituting Eq. (52)) into Eq. (32) in Methods, we obtain the distribution of Δz, that is,

$$p(\Delta z) \sim \Delta z^{-1+(1+2\mu/\gamma^2)}. \tag{55}$$

Up to now, we have obtained the distribution of Δz as a single component of $\mathbf{z}$. To get the distribution of $\|\Delta\mathbf{z}\|$, it is necessary to investigate the correlation between two components of $\mathbf{z}$ and $\mathbf{s}$. The correlation between s_1 and s_2 when both of them reach the stationary distribution is calculated to be,

$$\langle s_1(t_0+\mathrm{d}t)s_2(t_0+\mathrm{d}t)\rangle = \left[(1-\frac{\mu}{\tau_v})^2 + \frac{\gamma^2\mathrm{d}t}{\tau_v}\right]\langle s_1(t_0)s_2(t_0)\rangle. \tag{56}$$

In the case of $\mu > 0$ and $\mathrm{d}t$ an infinitesimal, we have $\left[(1-\mu/\tau_v)^2 + \gamma^2\mathrm{d}t/\tau_v\right] < 1$, which indicates that the correlation between s_1 and s_2 will converge to 0 exponentially, i.e., the correlation between these two components can be ignored under the condition of stationary distribution. Since $\Delta z(\tau_v) \sim s_{max}\tau_v$, it is straightforward that the correlation between Δz_1 and Δz_2 can be ignored as well. Hence, $\|\Delta\mathbf{z}\|$ (with $\|\Delta\mathbf{z}\| = \sqrt{\Delta z_1^2 + \Delta z_2^2}$) will follow the same power-law distribution as Δz (Eq. (55)), i.e.,

$$p(\|\Delta\mathbf{z}\|) \sim \|\Delta\mathbf{z}\|^{-1+(1+2\mu/\gamma^2)}. \tag{57}$$

This corresponds to Eq. (38) in Methods.

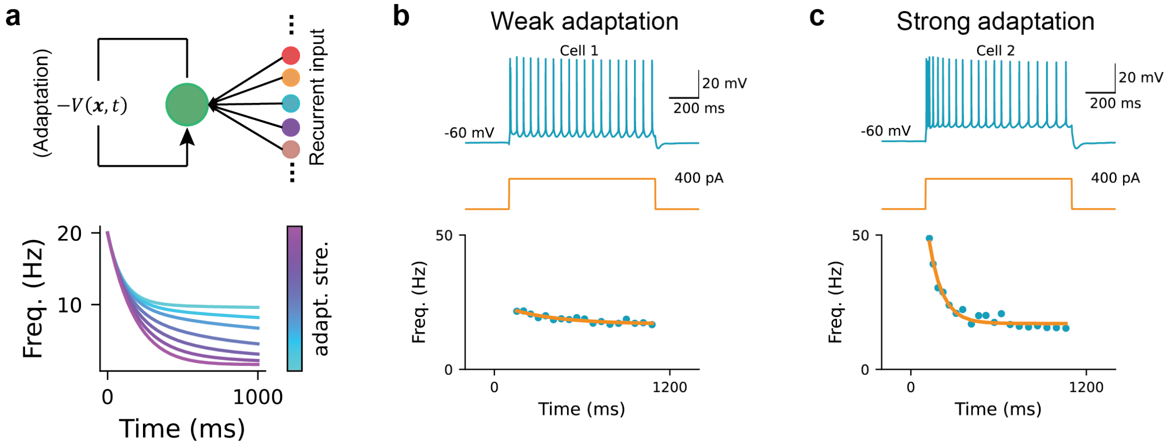

Figure S1: **Firing rate adaptation in the model and in a patch-clamp dataset from Yoshida et al.** (3) **(a)** Top: illustration of FRA which is a slow negative feedback modulation to cell firing. Bottom: cell firing frequency as a function of time when applying a constant input current. Different colors represent different adaptation strength. **(b)** Train (blue) of action potentials elicited in an in-vitro whole-cell patch-clamp cell during 1-second pulse injection (orange) showing weak FRA. Instantaneous firing frequency (blue dots) were shown at the bottom with an exponential fit (orange curves). **(c)** A cell showing strong FRA.

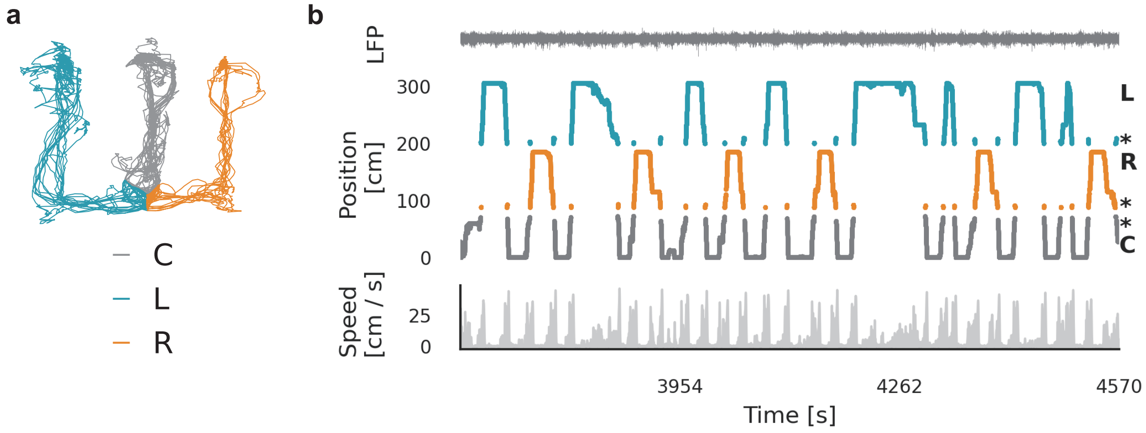

Figure S2: **The W-shape spatial alternation task from** (4)**.** **(a)**, the running trajectories from an animal during a recording session. Trajectories on the central arm, left arm and right arm are colored in grey, blue and orange, respectively. **(b)**, top panel: local field potential from a CA3 tetrode. Middle panel: the animal's trajectories alternating on the central-left-central-right-central arms over time, with the colors same as in **(a)**. Bottom panel: the animal's running speed over time.

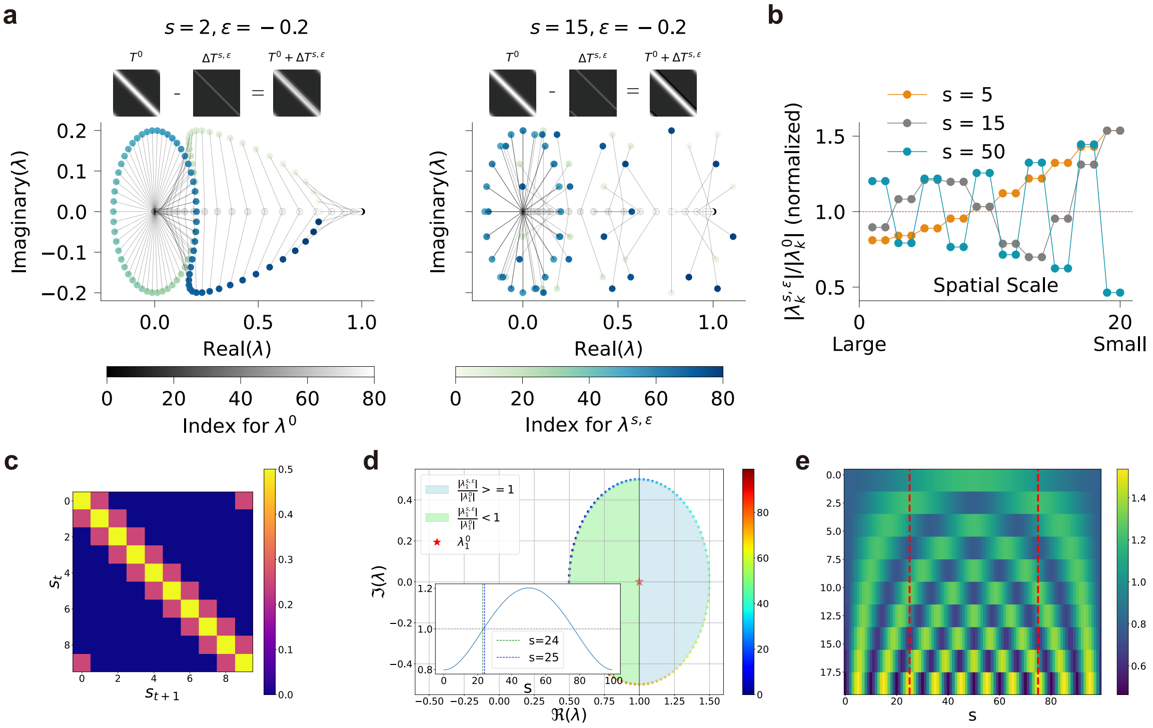

Figure S3: **The whole spectrum of the perturbed transition matrix. (a)**, Qualitative demonstration of perturbation to the eigenvalues following sub-diagonal perturbation to the transition dynamics. The perturbation patterns are heterogeneous with respect to perturbation offset, examples corresponding to $s = 2$ (left) and $s = 15$ (right) show vastly different distribution (symbols). grey bars link the perturbed eigenvalues with the original eigenvalues (without perturbation, lying on the real axis). **(b)**, rescaled eigenvalues (normalized by original eigenvalues) after the perturbation at different offsets (s=5: orange; s=15: grey and s=50: blue; top 20 eigenvalues are shown for better illustration). Theoretical analysis of oscillatory patterns in eigenvalue perturbations as a function of perturbation offsets. **(c)**, the "Lazy"-random walk transition matrix for analysis simplicity with 25% probability of transitioning to the left or the right location. **(d)**, perturbation of the second eigenvalue with different offset values under the simple "Lazy"-random walk scenario. $25 < s < 75$ gives amplified values of the eigenvalue (blue area) and $s < 25$ and $s > 75$ gives dampened values of the eigenvalue (green area). **(e)**, the complete spectrum of the eigenvalue rescaling under the simple "Lazy"-random walk scenario. x-axis represents the perturbation offset, each row is one rescaled eigenvalue (> 0 means amplifying; < 0 means dampening). Re-scaling of the first 20 eigenvalues are shown for demonstration. Red dashed lines mark the boundary of amplifying/dampening effect for the eigenvalue showed in **(d)**.

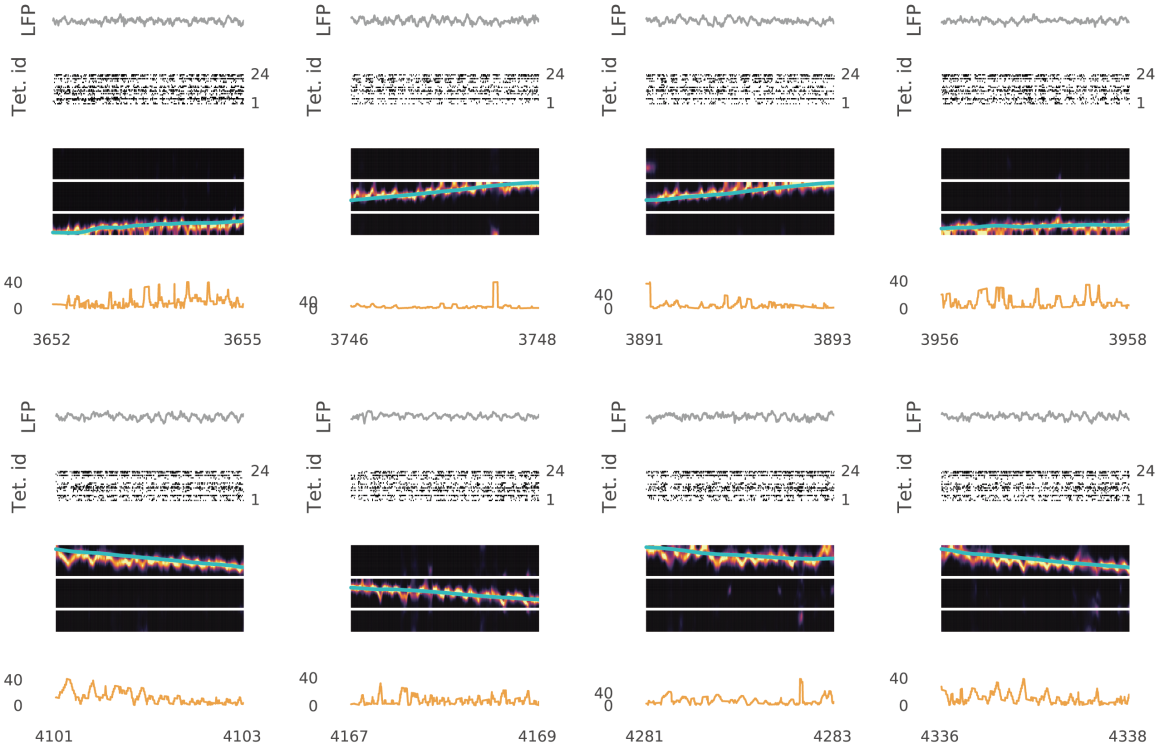

Figure S4: More examples of decoding theta sequences during running. From top to bottom in each example: the LFP signal from a CA1 tetrode; the multiunit activity; the posterior probability map; the offset distance between the decoded position and the actual position as a function of time (in seconds).

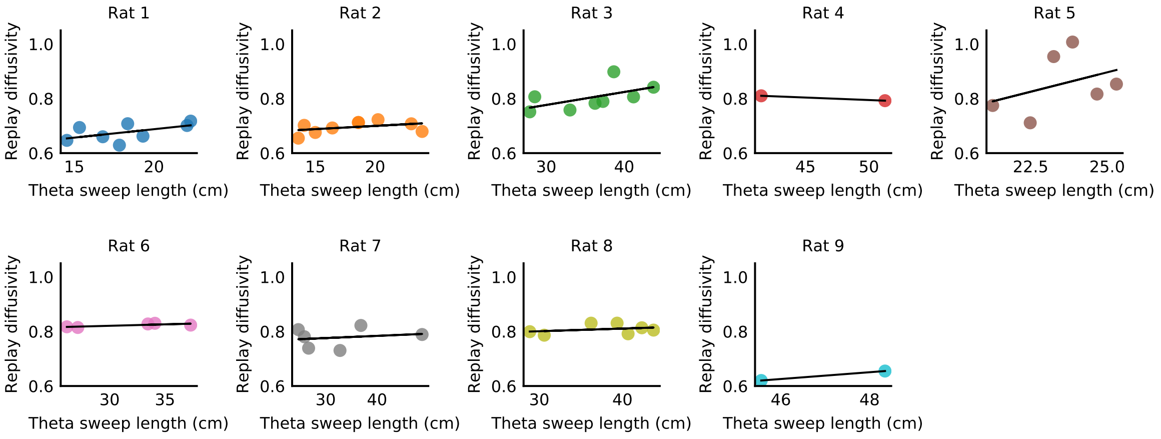

Figure S5: Correlation between replay diffusivity and theta sequence length across individual animals (9 in total). Each dot represents the diffusion exponent and the average length of theta sequences from a single recording day per animal. Note that while no significant correlation is observed, likely due to limited data points, there is a discernible trend suggesting a positive correlation.

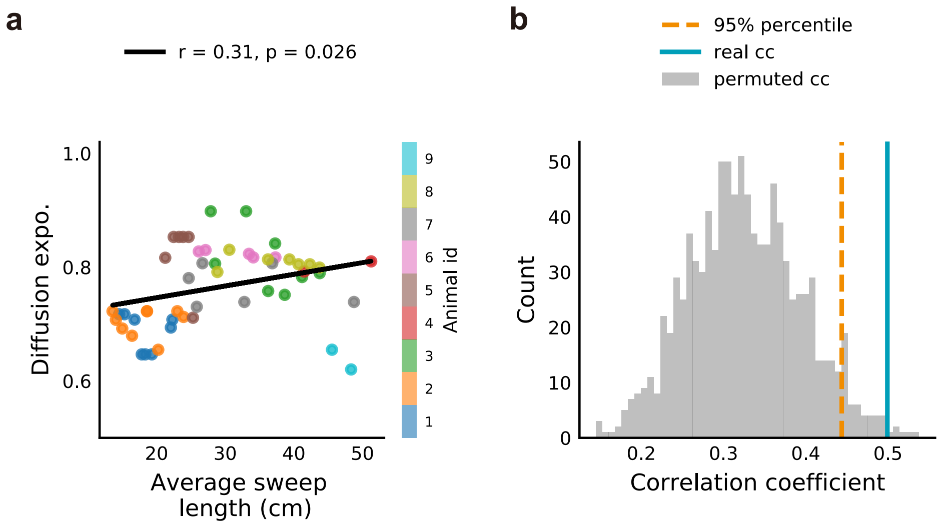

Figure S6: **Shuffling the replay diffusivity across recording days within the same animal.** (**a**), an example of the correlation between the theta sweep length and the shuffled replay diffusivity (by randomly sampling an replay diffusivity from another recording day of the same animal). (**b**), the histogram of the correlation coefficients between shuffled replay diffusivity and theta sweep length (1000 shuffles). The real correlation coefficient between replay diffusivity and theta sweep length is marked as the blue vertical line, and the 95% percentile of the shuffled correlation coefficients is marked as the orange dashed line.

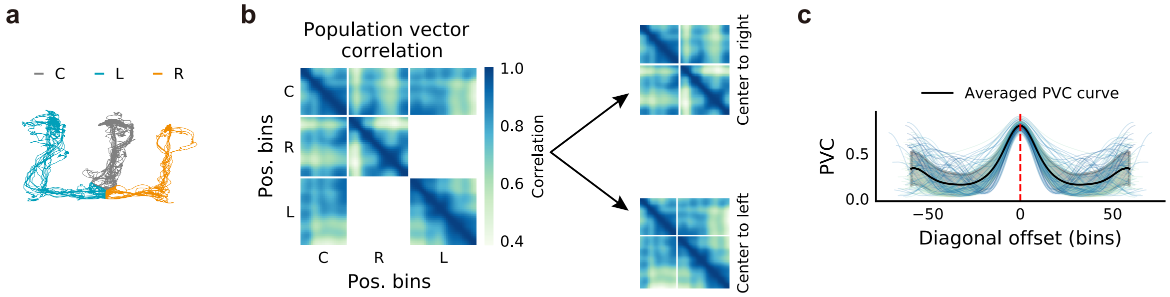

Figure S7: **Measuring the place field size index (population vector correlation analysis).** (**a**), the movement trajectory of an animal in one running session, with three arms coded by different colors. (**b**), the population vector correlation matrix shown in linearized spatial bins of center-right-left arms (left) and in separated center-right arm (top right) and center-left arm (bottom right). (**c**), the population vector correlation curve as a function of the diagonal offset of the correlation matrix. Each curve represents the correlation along the diagonal of one recording session, and the dark line marks the averaged curve. The correlation value is highest when correlation the population firing vector in the same spatial bin (auto-correlation), and slowly decays as the two spatial bins become further apart to each other (larger offset). Toward the end of the offset, there is an increase in correlation values, which may reflect an over-representation of reward locations at the end of the three arms or the geometrical similarity of the end of the three arms in the 2D space.

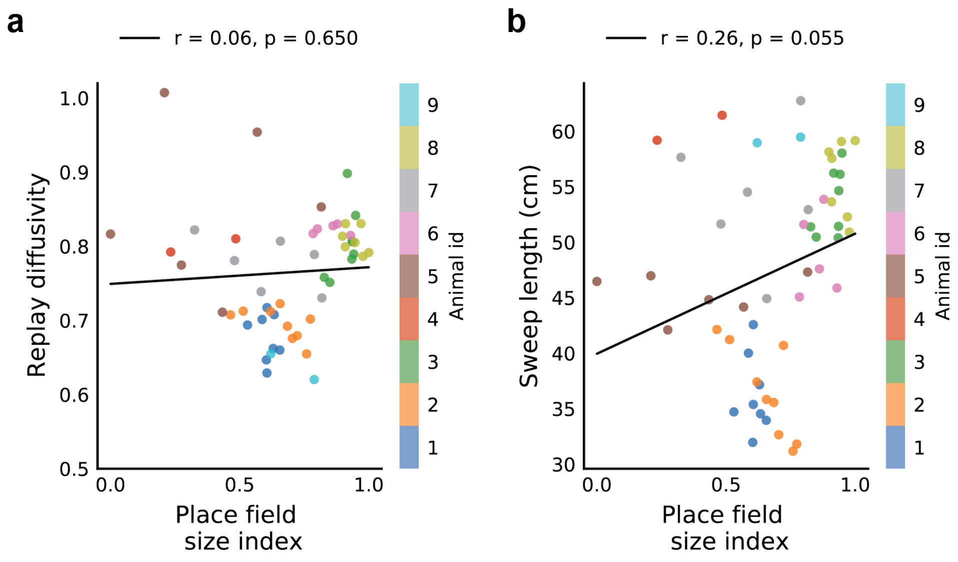

Figure S8: **Correlation of replay diffusivity and theta sequence length with place field size.** **(a)**, diffusion exponent as a function of the place field size index, with different colors representing data from different animals.. **(b)**, theta sequence length as a function of the place field size index.

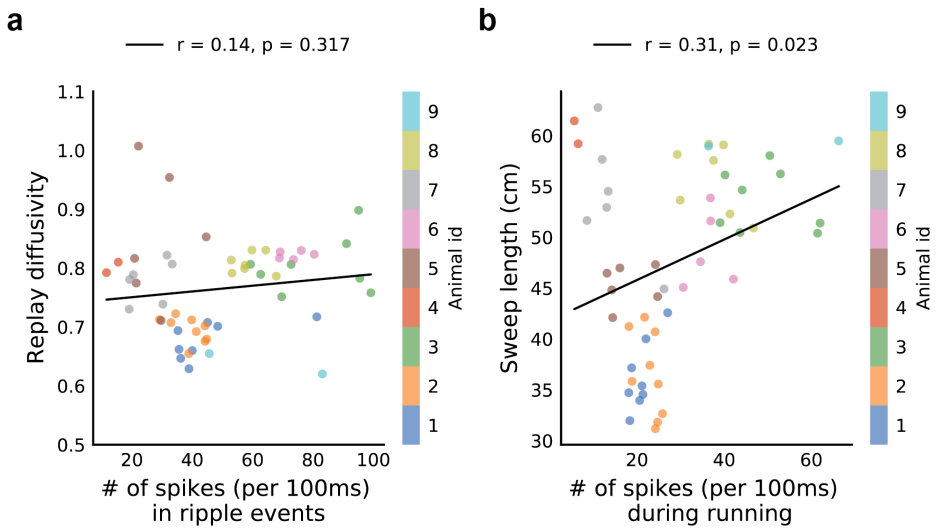

Figure S9: **Correlation of replay diffusivity and theta sequence length with the spike numbers participated in decoding.** **(a)**, correlation between replay diffusivity and the number of spikes (per 100 ms) participated in offline decoding. **(b)**, correlation between theta sweep length and the number of spikes (per 100 ms) participated in online decoding.

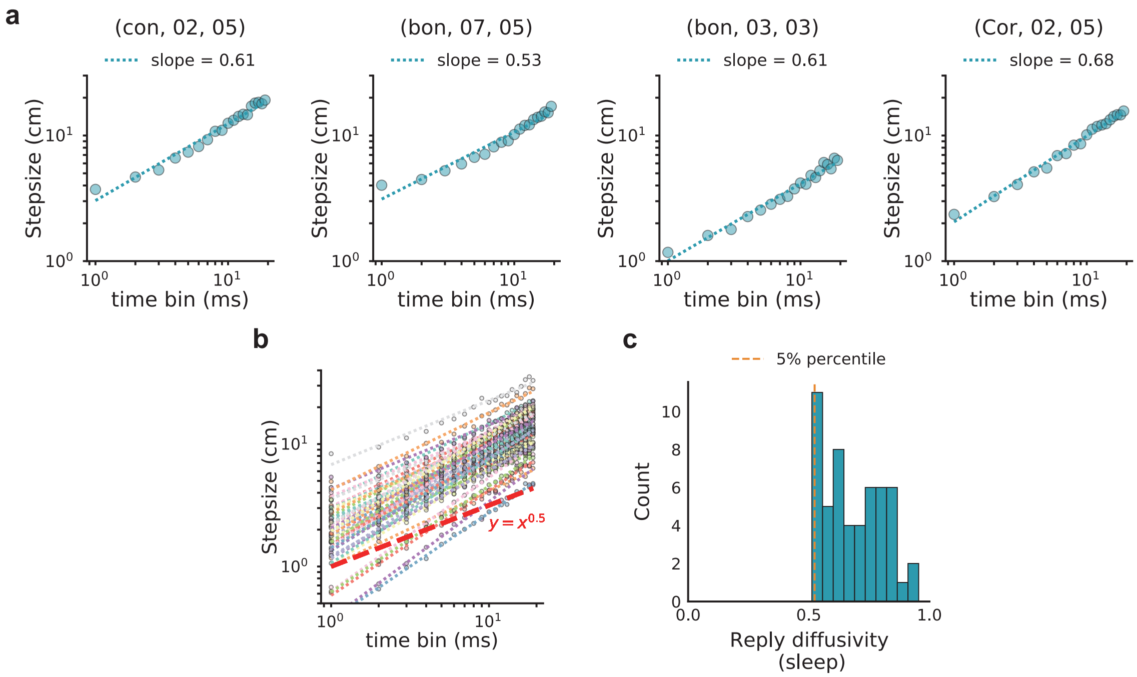

Figure S10: **Diffusivity values during sleep replay.** **(a)**, the log-log relationship between step size and time bin from four randomly selected sleep sessions. **(b)**, log-log plots from all valid sleep sessions. Each line represents one session (53 valid sessions in total). The red dashed line marks a typical Brownian-diffusion process with a slope of 0.5. **(c)**, histogram of diffusion exponents (one per sleep session) for sleep replay across all sessions and animals. The orange dashed line indicates the 5th percentile of the measured diffusivity values. Note that sleep replay is significantly larger than 0.5, diverging from the Brownian-diffusive dynamics.

References

[1] Rivers, R. *Path integral methods in quantum field theory* (Cambridge University Press, 1988).

[2] Lubashevsky, I., Friedrich, R. & Heuer, A. Realization of lévy walks as markovian stochastic processes. *Physical Review E* **79**, 011110 (2009).

[3] Yoshida, M., Jochems, A. & Hasselmo, M. E. Comparison of properties of medial entorhinal cortex layer ii neurons in two anatomical dimensions with and without cholinergic activation. *PLoS One* **8**, e73904 (2013).

[4] Karlsson, M., Carr, M. & Frank, L. Simultaneous extracellular recordings from hippocampal areas ca1 and ca3 (or mec and ca1) from rats performing an alternation task in two w-shapped tracks that are geometrically identically but visually distinct. *CRCNS. DOI: https://doi. org/10.6080/K0NK3BZJ* (2015).
